# An Innovative Approach for Retrieval of a Separated Intracanal File Using Guided Endodontics: A Case Report

**DOI:** 10.7759/cureus.77393

**Published:** 2025-01-13

**Authors:** Sachin Gupta, Ayushi Sharma, Shikha Jaiswal, Rudhra Koul, Shivali Tyagi

**Affiliations:** 1 Conservative Dentistry and Endodontics, Subharti Dental College and Hospital, Meerut, IND; 2 Conservative Dentistry and Endodontics, Genesis Institute of Dental Sciences and Research, Ferozpur, IND

**Keywords:** case report, cbct, fractured instrument, guided endodontics, instrument retrieval

## Abstract

A separated instrument in a root canal limits accessibility for chemo-mechanical preparation, making its retrieval imperative for a good prognosis. Among the numerous techniques available for instrument retrieval, guided endodontics is arguably the most predictable. This case report describes a stepwise approach for instrument retrieval using a surgical drill guided by a 3D-constructed acrylic template with the aid of cone beam computed tomography (CBCT). A 22-year-old male patient of North Indian origin presented with a chief complaint of discoloration and pain in tooth 21. Radiographic examination revealed poor obturation and fractured instruments in the middle third of the root canal. A diagnosis of a previously treated tooth with symptomatic apical periodontitis was made. After obtaining the patient’s consent, CBCT imaging and an intraoral scan were performed to generate Digital Imaging and Communications in Medicine (DICOM) and standard tessellation language (STL) files, respectively. These files were merged using Blue Sky Plan software (Blue Sky Bio, LLC; Grayslake, Illinois) to create a virtual drill path, which was subsequently used to fabricate a 3D acrylic guide, leading to the successful retrieval of the fractured instrument. Guided endodontics offers a predictable method for fractured instrument retrieval.

## Introduction

According to the American Association of Endodontics, over 15 million teeth undergo endodontic treatment annually. Endodontic treatment carries inherent challenges, one of which is the risk of instrument separation. During routine therapy, endodontic instruments may fracture at any stage due to factors such as flexural fatigue, torsional stress, or manufacturing defects. The reported incidence of intracanal fractures for endodontic instruments ranges from 0.25% to 6%. Effective disinfection and shaping of infected root canals require the removal of infected pulp tissue and any obstructions within the canal system. Instrument fracture can compromise the thorough shaping and cleaning of the root canal, potentially affecting the treatment's success and long-term prognosis [1].

Fracture rates for stainless steel (SS) hand instruments have been reported to range from 0.25% to 6% [2-5], while those of NiTi rotary instruments are reported to range from 1.3% to 10.0% [6,7]. Managing such incidents often involves retrieving the separated instrument, a process that is notably complex and heavily reliant on the clinician's skill and expertise [8].

Guided endodontics offers a promising and predictable solution for instrument retrieval [9]. This innovative technique employs a customized 3D-printed guide, designed using cone-beam computed tomography (CBCT). The guide is stabilized intraorally, allowing precise targeting of the separation site, as well as predefining the required angulation and depth for retrieval [10]. Guided endodontics, supported by computer-assisted treatment planning, significantly reduces the risk of perforations and other iatrogenic complications by ensuring a precise pathway for root canal access and instrumentation. Its applications include endodontic access cavity preparation, addressing pulp calcifications, performing osteotomies and apicectomies, and facilitating the removal of glass fiber posts.

This case report discusses the application of CBCT-guided endodontics to retrieve a fractured instrument from a previously obturated, symptomatic maxillary central incisor. The primary goal of this guided approach was to facilitate the predictable removal of the separated file using the mechanical action of a surgical drill, ultimately enhancing the prognosis of the case.

## Case presentation

**Figure 1:**
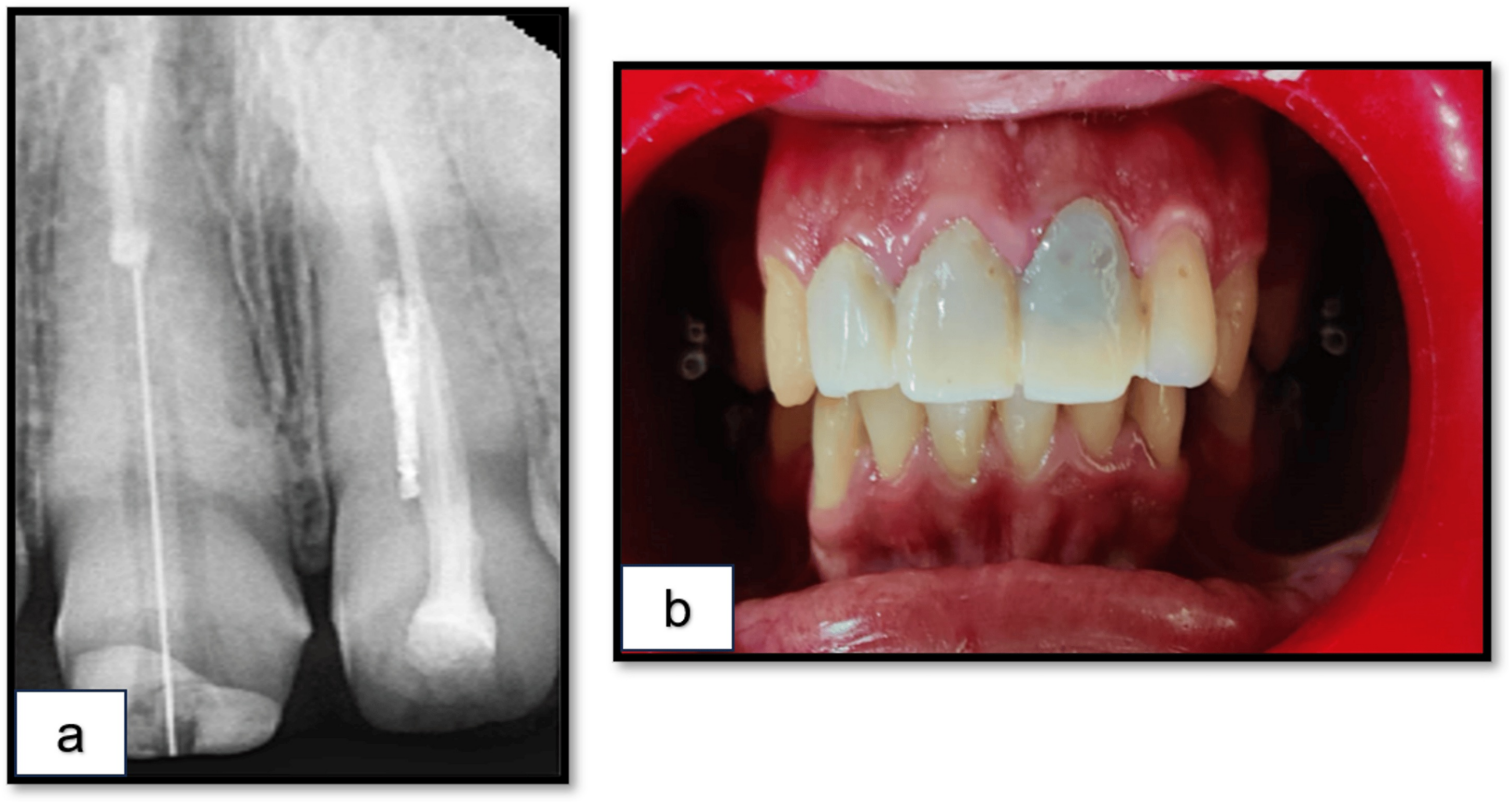
Preoperative findings at T=0 (initial presentation). (a) Radiograph showing a separated instrument in relation to tooth 21. (b) Clinical image displaying discoloration in relation to tooth 21.

A 22-year-old male patient of North Indian origin presented to the outpatient department of Conservative Dentistry and Endodontics with the chief complaint of discoloration and mild pain in the left upper front teeth. The patient’s past medical, family, and psychosocial history were non-contributory. Dental history revealed a previously root canal-treated anterior tooth with discolored artificial crowns. Clinical examination showed tenderness to palpation and percussion in relation to tooth 21. An intraoral periapical (IOPA) radiograph revealed root canal-treated teeth 21 and 11, with fiber post placement in tooth 11 and a poorly obturated tooth 21. A linear radiopacity was observed, indicating a fractured ProTaper F1 rotary instrument (Dentsply Sirona, Ballaigues, Switzerland) measuring approximately 6 mm, extending into the middle canal space (Figure 1a, b).

Using the aforementioned information, a diagnosis of a previously treated tooth with symptomatic apical periodontitis was made. A nonsurgical retreatment utilizing a CBCT-guided approach was planned, and informed consent was obtained from the patient after discussing all the pros and cons, including the economic aspects of the treatment.

Clinical procedure

**Figure 2:**
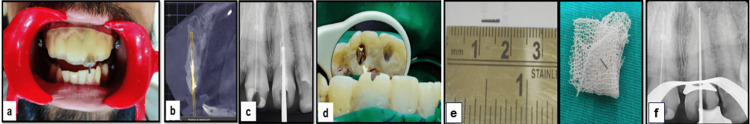
Operative procedures at T=1 week. (a) Try-in of the template. (b) Sagittal section of CBCT showing the virtual drill path. (c) Drill positioned adjacent to the separated instrument. (d) Retrieval of the file. (e) Retrieved file fragment. (f) Working length determination.

A limited CBCT scan (Orthophos SL 3D, Dentsply Sirona, Charlotte, North Carolina) was performed with exposure parameters set at 80 kV, 3.0 mA, and 17.5 seconds. Following analysis of the axial, sagittal, and coronal slices, an intraoral optical scan of the tooth surface was obtained. The CBCT data and the optical scan were exported as DICOM and STL files, respectively. These files were then imported into the implant planning software Blue Sky Plan (Blue Sky Bio, LLC; Grayslake, Illinois) and merged to create a virtual drill path (Figure 2a).

Fabrication of virtual guide

A virtual guide base was generated with a guided tube matching the dimensions evaluated using CBCT. The CBCT data were superimposed with STL files to fabricate an acrylic guide using the Formlabs 2 3D printer (Formlabs Inc., Somerville, Massachusetts). A trial of the virtual guide was conducted on the patient to assess fit and stability (Figure 2b). The 3D-printed guide (Formlabs 2, material: Dental SG) with a transparent acrylic sleeve was fabricated with a thickness of 3.5 mm and a height of 5 mm. The outer diameter of the sleeve was designed to be 0.1 mm larger than the inner diameter, corresponding to the diameter of the chosen drill.

Instrument removal

The procedure was performed under an 8X magnification surgical microscope. A 1-mm-diameter trephine was attached to a contra-angled handpiece, positioned within the guide, and operated at 500 rpm in an anticlockwise direction. The resin guide directed the progress of the trephine within the root canal until the target point, which was radiographically confirmed (Figure 2c). The trephine drill advanced stepwise until 2 mm of the separated instrument's head was exposed. At each step, the guide was removed, and the root canal was irrigated with sodium hypochlorite and normal saline to clear debris, followed by cleaning the trephine.

The separated instrument was eventually loosened by the mechanical action of the drill (1.3 mm diameter, Neodent, Curitiba, PR, Brazil) (Figure 2d) and removed during irrigation (Figure 2e) [11]. Working length (WL) was re-established (Figure 2f), and shaping was performed using the crown-down technique up to F3 Protaper Gold (Dentsply, Ballaigues, Switzerland), combined with circumferential filing. The irrigation protocol included 2 ml of 3% sodium hypochlorite (Pyrax Polymars, Roorkee, India), followed by 17% EDTA (Waldent Innovations Pvt Ltd, New Delhi, India) for three minutes.

**Figure 3:**
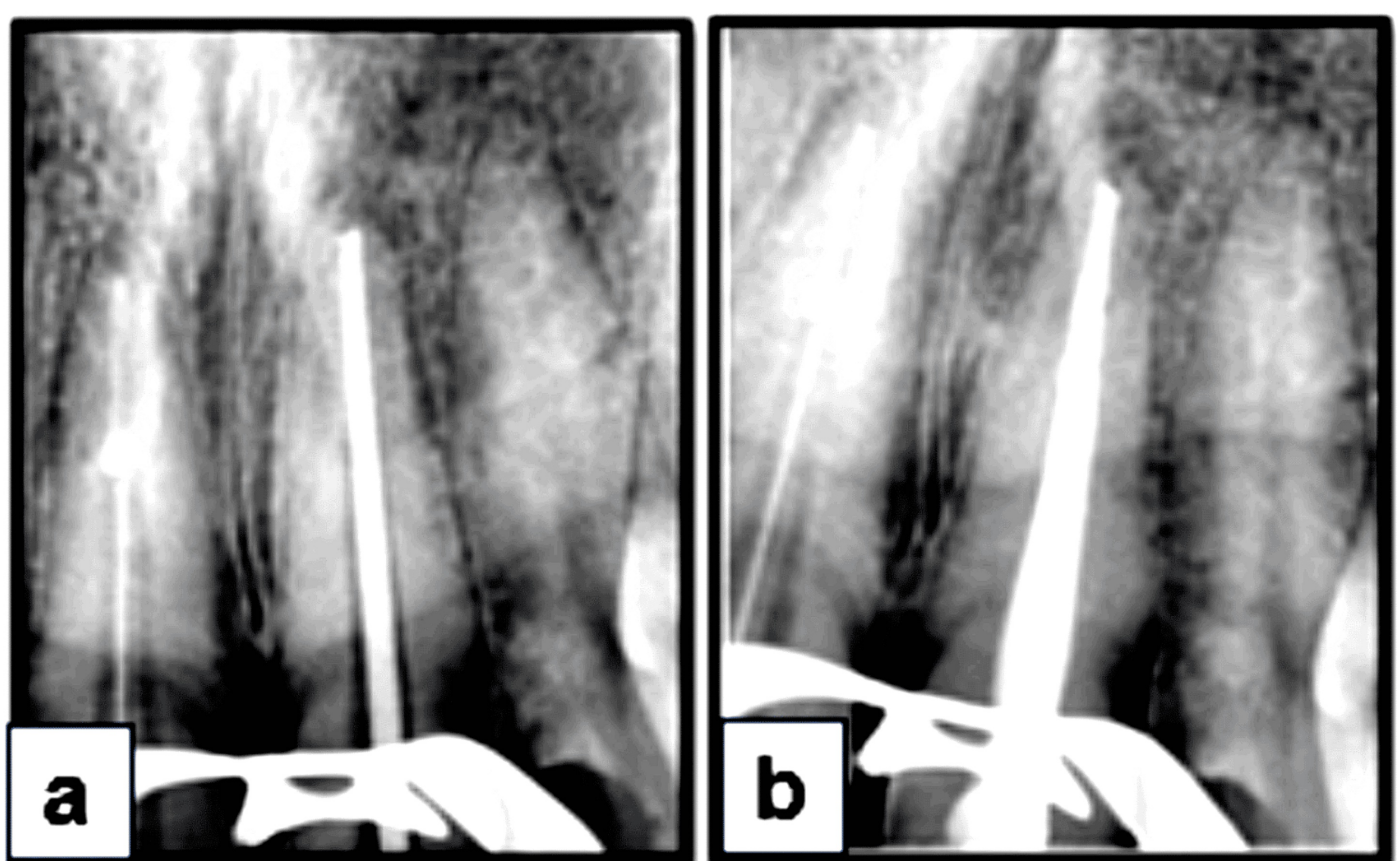
At T=2 weeks: Obturation and post-endodontic restorations. (a) Master cone radiograph. (b) Post-operative radiograph showing obturation in relation to tooth 21.

Calcium hydroxide was placed as an intracanal medicament (ICM) for two weeks. A study by Donyavi et al. demonstrated that calcium hydroxide combined with 0.2% chlorhexidine significantly reduced aerobic, anaerobic, and *E. faecalis* colony counts over two weeks [12]. At the second appointment, when the patient was asymptomatic, obturation was completed using the downpack technique. The plugger was repeatedly introduced to 3-5 mm from the apex, utilizing the Calamus system (Dentsply Sirona). The middle and coronal thirds of the canal were then backfilled using the gutta-percha injection technique (Figure 3a, b).

**Figure 4:**
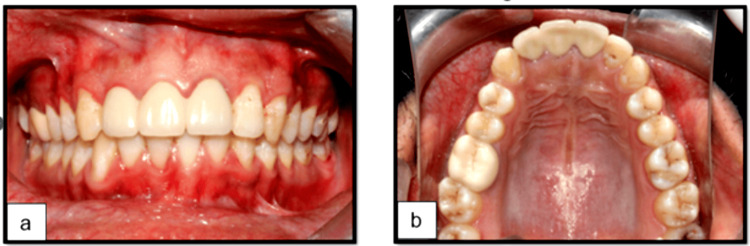
At T=1 month: Provisional restoration with temporary crowns. (a) Labial view. (b) Occlusal view in relation to tooth 21.

Post-endodontic composite restoration was completed, and a temporary crown was fabricated and placed one month later, as the patient is currently undergoing orthodontic treatment (Figure 4a, b).

**Figure 5:**
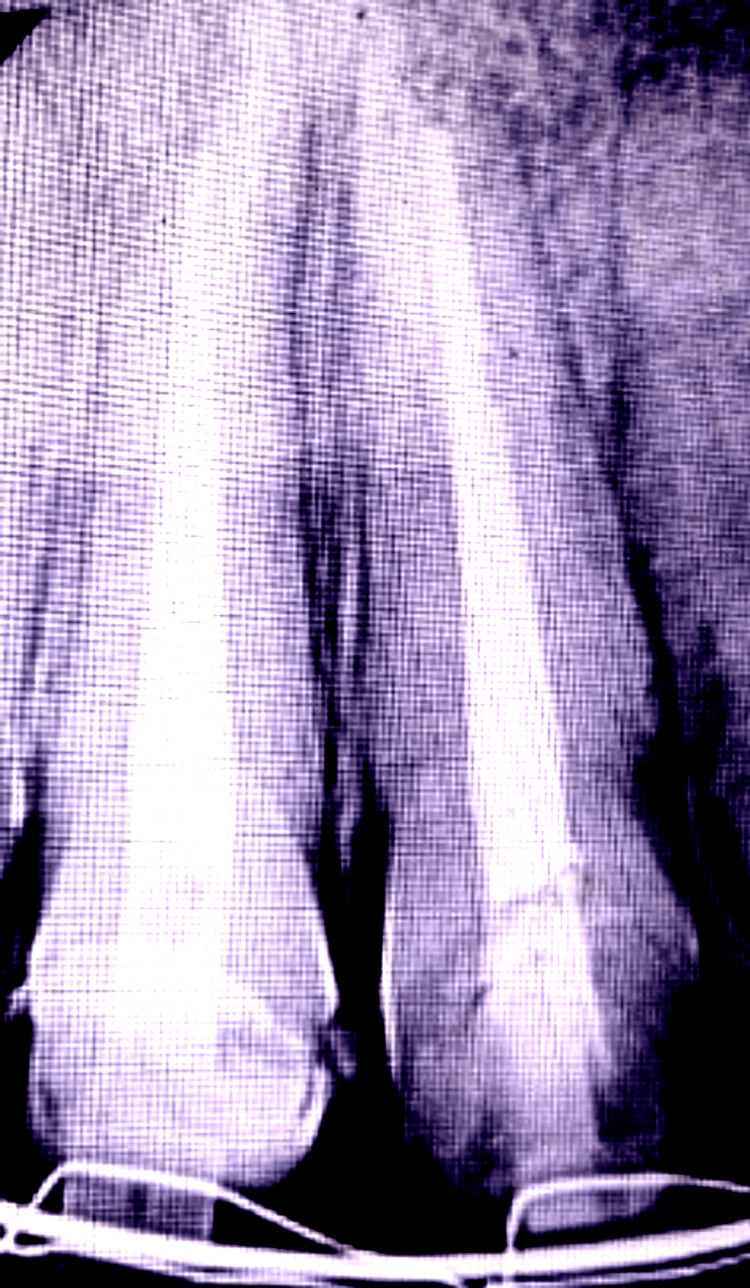
At T=1 year: Follow-up. Post-operative radiograph showing evidence of periapical healing in relation to tooth 21.

At the one-year follow-up, the tooth remained asymptomatic, with radiographic evidence of periapical healing (Figure 5).

Operator-based outcomes

Guided endodontics enables the retrieval of separated instruments with minimal dentin loss, allowing effective cleaning of the root canal system and the removal of residual microflora that may have persisted due to the presence of a foreign body. The judicious use of irrigants and the placement of ICMs contribute to the successful resolution of apical periodontitis.

Patient-based outcome

The patient became asymptomatic within one week of treatment, with no tenderness to percussion or pain reported.

## Discussion

Fractured instruments present a significant challenge in endodontic practice, as they hinder effective disinfection of the root canal system [13]. Attempts to bypass or retrieve these instruments often lead to complications such as perforation, canal stripping, or excessive canal enlargement, all of which can adversely impact the prognosis [14]. Research shows that removing fractured instruments from the middle or apical third of the root canal often compromises the tooth's mechanical integrity due to the loss of tooth structure associated with conventional retrieval techniques [15,16]. While ultrasonic tips are commonly used as a conservative option, they may result in adverse effects, such as dentinal microcracks or heat generation, which can harm periodontal tissues [17].

In contrast, CBCT-guided techniques offer a more predictable approach to fractured instrument retrieval [9]. CBCT provides three-dimensional visualization of anatomical structures, surpassing the capabilities of intraoral, panoramic, and cephalometric imaging. By processing projection data, CBCT generates interrelated images in axial, sagittal, and coronal planes. It also offers several advantages, including reduced radiation exposure, enhanced image quality compared to conventional radiographs, affordability, and minimal heat production.

This method involves creating a surgical template using CBCT data, optical scans, and planning software [10]. By enabling straight-line access with minimal dentin loss, guided endodontics reduces intraoperative errors and preserves tooth structure [14]. Additionally, it minimizes chairside time and the need for repeated radiographic exposures, enhancing patient comfort and compliance. Despite its advantages, this technique is primarily applicable to straight canals, which is a notable limitation. Promising results have been demonstrated in managing complex endodontic cases, such as pulp canal obliteration [18], dens invaginatus [19], and fiber-post removal [20]. However, there is limited evidence regarding its use for fractured instrument retrieval. Further research is needed to explore its potential for removing file fragments obstructing access to the apical third of the root canal.

The clinical course began at T=0, when the patient presented with complaints of discoloration and pain. After obtaining informed consent, instrument retrieval using a guided endodontic approach was planned. Intraoral scanning and CBCT imaging were performed during the initial visit, and both scans were sent to the lab for guide fabrication. At T=1 week, instrument retrieval was successfully performed with the 3D-printed guide, followed by the establishment of patency and working length determination. ICM was placed for two weeks. At T=2 weeks, obturation and post-endodontic restoration were completed. At T=1 month, a temporary crown was placed until the completion of orthodontic treatment. At T=1 year, a follow-up radiographic evaluation showed evidence of periapical healing.

## Conclusions

This report describes the removal of a separated instrument using a novel approach that combines computer-guided planning and a resin guide. The guided endodontic technique reduces unnecessary loss of dental structure, optimizes efficiency by minimizing chairside time, and improves the operator’s confidence during the procedure. Guided endodontics emerges as a predictable and minimally invasive method for retrieving fractured instruments in straight canals, particularly in retreatment cases.
